# Scalable Emitter Photonic Integrated Circuits on an Open‐Access Indium Phosphide Foundry

**DOI:** 10.1002/nap2.70268

**Published:** 2026-08-27

**Authors:** Durvasa Y. Gupta, Axel Schoenau, Hendrik Boerma, Shahram Keyvaninia, Patrick Runge

**Affiliations:** ^1^ Fraunhofer‐Institut für Nachrichtentechnik Heinrich‐Hertz‐Institut Berlin Germany

## Abstract

We present an easy access prototype solution using Fraunhofer‐HHI's open‐access indium phosphide photonic integrated circuits (PIC) platform. The process design kit enabled generic multiproject wafer process co‐integrates low‐loss passive components with gain, modulation, and detection. A high‐speed 80 GBaud traveling‐wave Mach–Zehnder modulator (MZM) is a key building block for emitter PICs. Accordingly, we demonstrate wafer‐scale, all‐electrical predicing tests using an MZM integrated with an on‐chip distributed‐feedback laser and photodiode. To showcase the platform's breadth, we highlight three emitter PICs. A widely tunable electro‐optic comb generator with a 20‐dB comb bandwidth of 480 GHz, a 670 MHz low‐repetition‐rate extended‐cavity mode‐locked laser, and a monolithic tunable ring laser with a 40 nm single‐mode tuning range and a 8.2 kHz intrinsic linewidth. All three PIC examples enable data‐rate scaling in optical communication by either increasing the number of channels or by increased symbol rate. Lastly, we present a convenient packaging solution that shortens lead time for prototype‐testing.

## Graphical abstract

An open‐access indium phosphide platform with high‐speed 80 GBaud modulators delivers versatile emitter PICs. By showcasing a widely tunable optical frequency comb generator, a 672 MHz record low‐repetition‐rate mode‐locked extended cavity mode locked laser, and monolithic InP tunable laser with a narrow linewidth of 8.2 kHz, this work presents a pathway to scaled optical communications. Advanced wafer‐scale electrical testing and packaging solutions optimize prototype development.

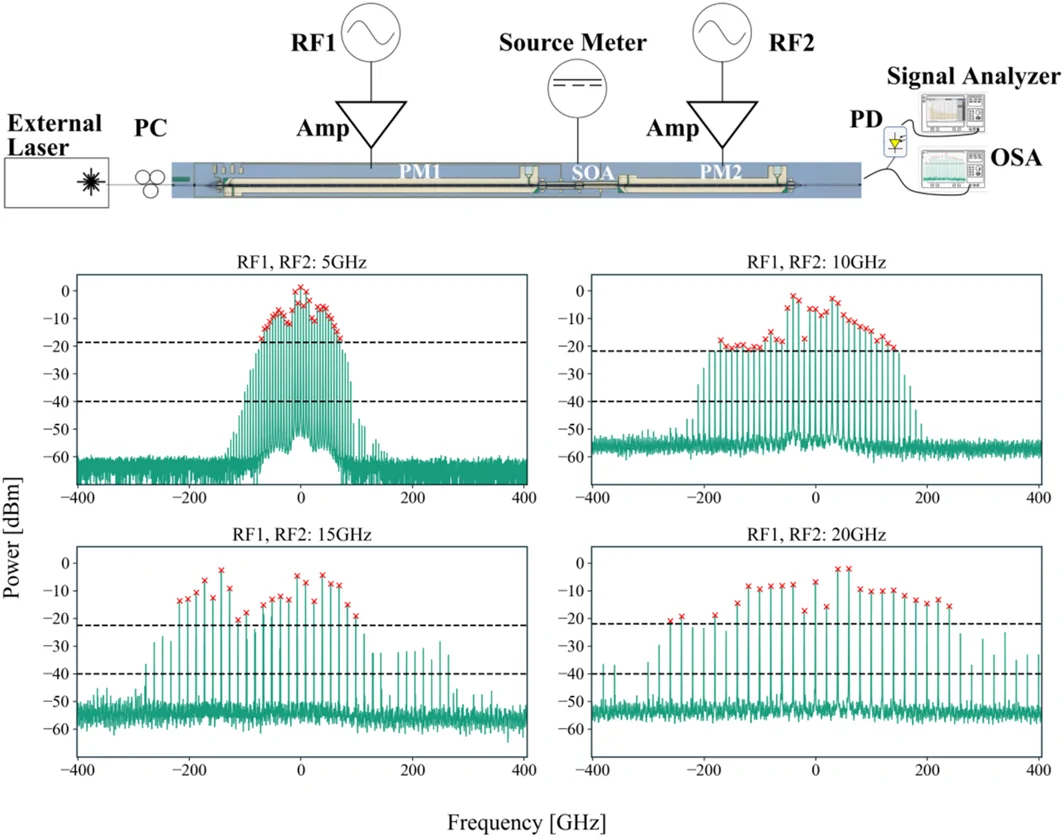

## 1 Introduction

Rapid growth in data‐center interconnects [1], long‐haul coherent transport [2], and emerging sensing [3] and microwave‐photonics applications [4] are fueling demand for compact, power‐efficient, and manufacturable photonic integrated circuits (PICs). Among available material platforms, indium phosphide (InP) remains unique in offering gain, high‐speed modulation, photodetection, and low‐loss passive routing across the C‐band (1530–1565 nm), thereby enabling monolithic transceivers and advanced sources on a single chip. Open‐access generic foundries, in turn, have transformed PIC development by providing standardized building blocks and scheduled multiproject wafer (MPW) runs that reduce design cycles and lower cost.

Building on Fraunhofer HHI's (FhG‐HHI) prior multiproject wafer (MPW) platform report [5], which covered lasers, semiconductor optical amplifiers (SOAs), electro‐absorption modulators (EAMs), photodiodes (PDs), polarization handling, spot‐size converters, and hybrid I/O, we summarize the recent addition of traveling‐wave Mach–Zehnder modulators (MZMs) reported previously in ref. [6]. The MZMs are based on the quantum‐confined Stark effect (QCSE) with low drive voltage and large bandwidth, enabled by an added modulator multiquantum well (MQW) stack and optimized metallization.

Wafer‐level testability is critical for screening and yield control in large‐scale manufacturing. By co‐integrating gain sections on‐chip, optical signals can be generated and detected without external sources, enabling test‐before‐dice procedures that reduce cost and accelerate iteration. We demonstrate this capability by all‐electrical characterization of a high‐speed MZM, a crucial component in emitter PICs, and a high‐speed PD driven by an integrated distributed‐feedback (DFB) laser.

To illustrate the platform's breadth, we highlight three representative emitter PICs each with distinct applications: A widely tunable electro‐optic optical frequency comb generator (OFCG), that cascades phase modulators with on‐chip gain, with comb spacing tuning between 5 and 20 GHz and center wavelength tuning of 35 nm; a monolithic extended‐cavity mode‐locked laser achieving a record‐low on‐chip repetition rate of 670 MHz; and a monolithically integrated ring‐resonator tunable laser with an intrinsic linewidth of 8.2 kHz and a 40 nm tuning range.

The first two emitter PICs are OFCGs that respond to the need for compact, low‐power, and cost‐effective dense comb sources vital for a broad range of applications, including microwave photonics [7] and spectroscopy [3]. The first emitter PIC targets tunability for microwave photonics applications, where tunability in wavelength and free‐spectral range (FSR) are crucial [7], and is based on electro‐optic modulation frequency comb generation. The second emitter PIC responds to the need for an OFCG with sub‐GHz comb line spacing for high‐resolution spectroscopy and relies on an extended cavity mode‐locked laser architecture. The third emitter PIC is a widely tunable laser with a narrow linewidth which is a crucial building for high‐speed coherent communications [8].

All three emitter PICs presented demonstrate data‐rate scaling in optical communication by either increasing the number of available channels or by enabling higher symbol rates per channel. The comb generator and mode‐locked laser both produce dense optical frequency combs, providing multiple equally spaced wavelengths that can be individually modulated for parallel data transmission (scale out). The tunable ring laser offers wide agile wavelength tuning and narrow linewidth, which supports higher order modulation formats and allows flexible assignment of channels, maximizing spectral efficiency (scale up). By integrating these devices with high‐speed modulators and wafer‐scale testability, the foundry enables scalable solutions that address the growing bandwidth requirements of modern optical networks.

In the final section, we present FhG‐HHI's packaging solution, integrated with its foundry service: standardized packaging and layout templates, plus modular RF/DC/optical interfaces (RFconnect, RF Evaluation Board, and PIConnect) that implement open testing standards, enabling short cycle times, consistent measurement workflows, and high technology readiness (TRL) prototypes.

## 2 Process Overview

As reported in ref. [6], the FhG‐HHI generic foundry process integrates active and passive elements on semi‐insulating InP, combining optical amplification and electro‐absorption stacks with low‐loss waveguides. To enable high‐speed phase modulation with minimal insertion loss, an additional InGaAsP/InP multiquantum‐well stack is introduced for the modulator, with its absorption edge tailored to minimize transmission loss. The crystal orientation is adjusted from [110] to [1$\bar{1}$0] to enhance additive electro‐optic effects for TE polarization.

Active and passive waveguides are connected through butt‐joint regrowth steps that minimize reflection losses. Dry and wet etching define active mesas, routing waveguides, polarization elements, and spot‐size converters. The initial epitaxy comprises a diluted waveguide, an n‐doped buffer, and active layers formed by eight strained‐compensated InGaAsP MQWs centered near 1540 nm. DFB gratings and distributed Bragg reflector (DBR) gratings are patterned by electron‐beam lithography and etched via reactive ion etching. DFB gratings integrated within the gain MQW stack achieve a coupling coefficient of approximately 550 cm^−1^. Receiver areas are selectively etched and passively routed, followed by passivation for electrical isolation and metallization optimized to reduce parasitic capacitance and series resistance. With the e‐beam lithography minimum feature size down to 90 nm can be achieved. All epitaxial layers are grown using metal‐organic vapor phase epitaxy (MOVPE) in a production‐scale Aixtron AIX 2800‐G4 reactor. A schematic illustration of the epitaxial layer structure is provided in Figure 1.

**FIGURE 1:**
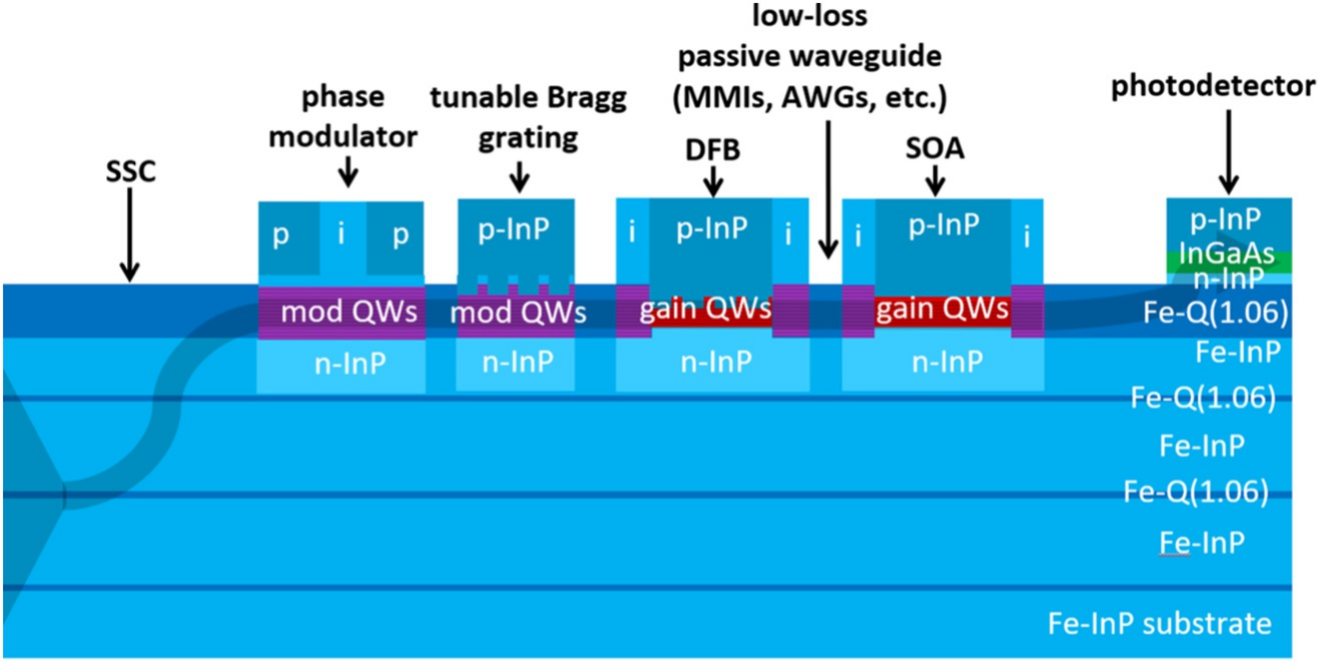
Schematic showing the epitaxial layers of the generic foundry process. Reproduced from ref. [6].

The monolithic integration of InP PICs provides superior thermal management compared to hetero‐ and hybrid approaches. Fabricating all components on a single substrate ensures excellent thermal uniformity, which stabilizes wavelengths and preserves signal integrity. In this work, the bare InP chips are placed directly onto a copper heat‐sink block governed by an external thermo‐electric cooler (TEC) and a closed‐loop proportional‐integral‐derivative (PID) temperature controller. This benchtop setup actively stabilizes the substrate base temperature to within ± 0.01°C of the setpoint (usually 20°C), ensuring reproducible measurements.

## 3 All‐Electrical Testing of an MZM

For this characterization, the DFB laser is biased at approximately 100 mA, the voltages applied across the lower and upper arms of the MZM (labeled “Lower electrode” and “Upper electrode”) are swept from 0 V to −12 V, and the photodiode is biased at −2 V. The resulting extinction ratio (ER) map is plotted in Figure 2b. The *V*_π_ can be calculated to be 2.33 V (denoted by black arrow in Figure 2a) for a 3 mm long MZM at −8 V bias giving a *V*_π_*L of 0.7 V‐cm, with a max. ER of > 24 dB. Here, the ER is limited by the light propagating in the substrate mode and, light coupled into a single mode fiber eliminates the substrate mode extensively and as already mentioned in ref. [6], a higher ER up to 31 dB is measured using an external PD.

**FIGURE 2:**
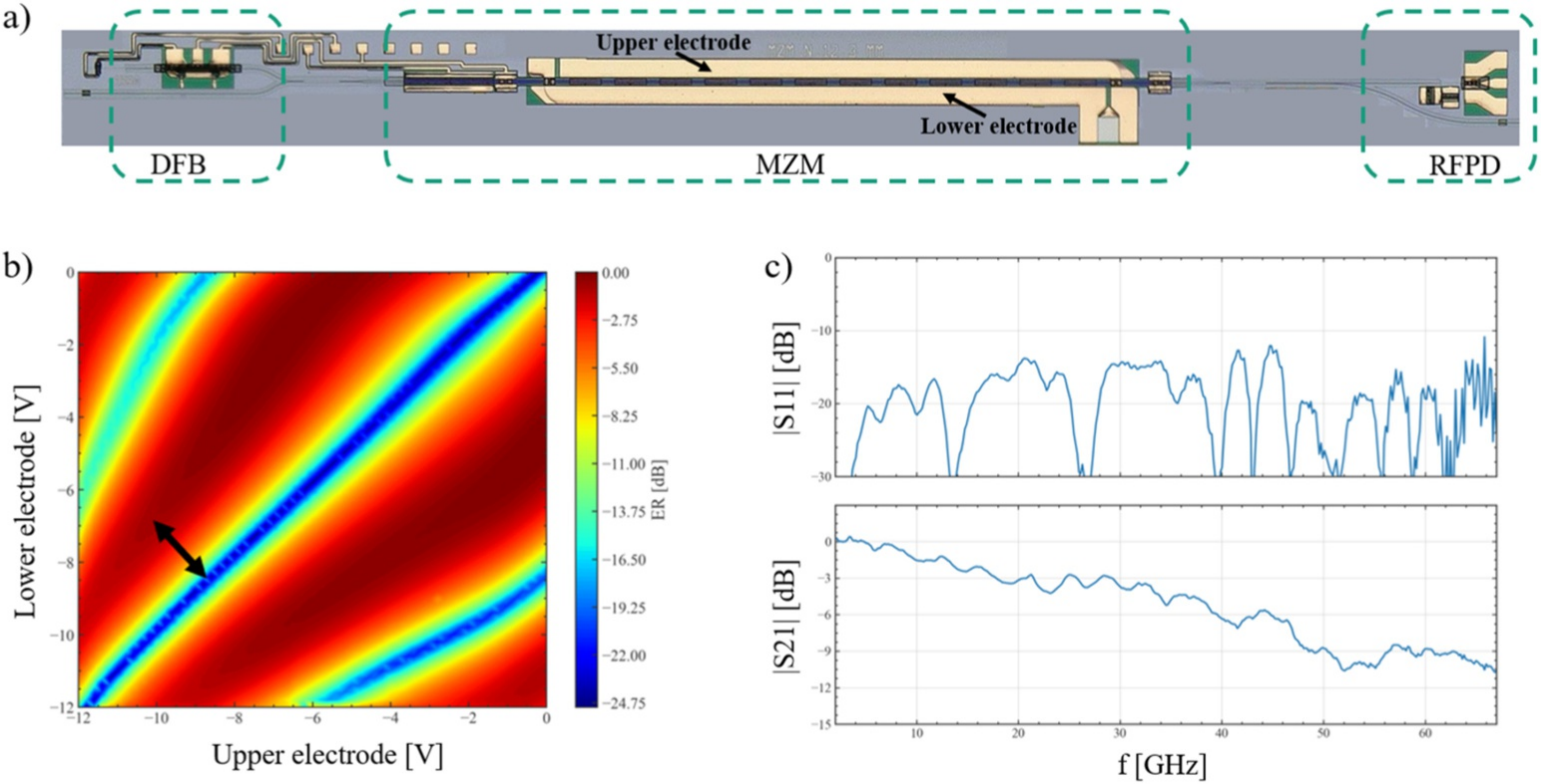
All electrically measured (a) microscopic image of the MZM test structure. (b) DC‐extinction ratio map. (c) All electrical small signal response of a 3 mm long MZM + RFPD.

Small‐signal frequency response is measured with a light‐component‐analyzer operated in vector network analyzer mode as the optical component of the instrument is not required for co‐integrated testing. The MZM is biased at −8 V and the photodiode at −2 V. Because the MZM and high‐speed PD constitute cascaded RF components, the effective 6‐dB electro‐optic bandwidth is the relevant figure of merit. From Figure 2c, it can be seen that the S_11_ is lower than −10 dB until 67 GHz which indicates 50 Ω impedance matching. From S_21_, the f_6dB_ of the 3 mm long MZM + high speed PD is 40 GHz, consistent with individual device performance. This demonstrates that all‐electrical testing yields actionable data for screening and process refinement. For the sake of completion, the large signal characterization (measured with an external PD) of the MZMs is reproduced here from ref. [7]. Figure 3 shows the large signal characterization of the MZM with a 5 mm long active section for 64 Gbps NRZ, 80 Gbps NRZ, and 24 GBaud PAM4. No preequalization or postdigital processing is used in the measurement. The extinction ratios are 9.3 dB, 8, and 5.5 dB, respectively (For detailed information on the characterization, please refer to ref. [6]).

**FIGURE 3:**
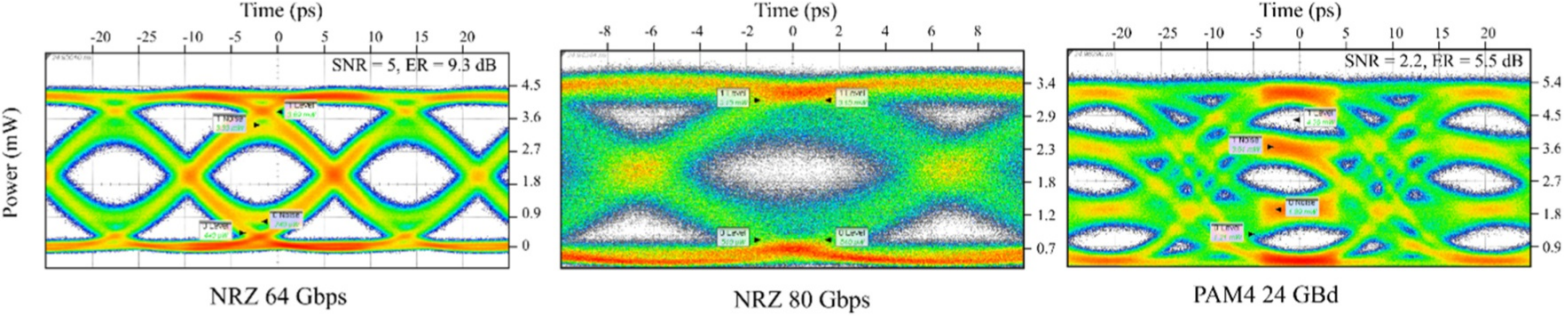
Optical eye diagrams for a 5 mm long MZM. Reproduced from ref. [6].

Electrical characterization offers several advantages for chip testing. It enables rapid evaluation of chips, allowing for high throughput and efficient processing. Since no optical alignment is required, the procedure is streamlined and saves valuable time. Automation across the wafer is straightforward, further enhancing efficiency. This approach is particularly suitable for photonic integrated circuits (PICs) that do not require optical access during testing.

However, there are notable limitations. Electrical characterization can only be performed after metallization, being one of the last steps before backend processing. This restricts the possibility of identifying issues earlier in the process, where intervention would be more impactful. Additionally, only coupled characteristics can be assessed, making it difficult to isolate and evaluate the performance of individual components. Nevertheless, in the case of PICs, the overall circuit performance is often more relevant. Finally, the quality of the chip facets after cleaving, a critical parameter for selecting good dies, remains unassessed. Therefore, additional visual or optical characterization is required to fully qualify the chips.

Components that can be tested using this method include, but not limited to, DFB lasers, MZMs, high‐speed PDs, balanced PDs, EAMs, DBR lasers, and sampled grating DBR lasers. The key requirement is the presence of a laser for light generation and a photodiode for detection. In this setup, the circuit's overall performance is inherently linked to the characteristics of the laser and the PD, making it difficult to assess components separately. This method is particularly suitable when the laser and PD are integral to the application or are implemented in a monitoring output, such as the MZM presented in this section.

To contextualize the performance of the FhG‐HHI's InP foundry platform, a comprehensive quantitative comparison is conducted against another open‐access monolithic InP MPW foundry platform. Restricting this benchmarking strictly to monolithic InP foundries ensures a rigorous evaluation. This methodology isolates architectural and design advantages without confounding variables, such as the severe coupling overheads, material interface mismatch, and fabrication complexities inherent, to hybrid or heterogeneous silicon integration. As summarized in Table 1, the FhG‐HHI InP MPW platform exhibits highly competitive performance metrics across all critical components, including active gain mediums, passive routing, high‐speed modulation, and detection blocks. Beyond the basic building blocks, the emitter PICs presented in the following sections will showcase the platform's breadth and versatility.

**TABLE 1:** Comparative benchmarking of the FhG‐HHIs InP foundry platform against an open‐access monolithic InP foundry.

Building block	Parameter	FhG‐HHI	Smart photonics [9, 10]
Waveguide (deeply‐etched)	Propagation loss	2 dB/cm	2 dB/cm
Spot‐size converter	Edge‐coupling loss (9 μm MFD)	1.5 dB	a
Distributed feedback laser	Threshold current	10 mA	b
	Tuning range	4 nm	b
	Output power	10 mW @50 mA	b
	Small signal bandwidth	20 GHz @ 100 mA	b
	Wall plug efficiency	5%–8%	b
Distributed Bragg reflector laser	Tuning range	5 nm	4 nm
	Output power	10 mW	10 mW
	Linewidth	< 1 MHz	< 1 MHz
Semiconductor optical amplifier	Gain (dB/mm)	27	30
	Saturation output power (dBm)	13	15
	Time constant	< 1 ns @ 100 mA	a
Mach–Zehnder modulator	*V*_π_*L	0.7 V‐ cm	0.7 V‐ cm
	Modulation bandwidth*Length	160 GHz‐mm	134 GHz‐mm
DC photodiode	Responsivity	0.8 A/W	> 0.7 A/W
	Dark current	2 nA @ −2 V bias	25 nA @ −2 V bias
High speed photodiode	3‐dB bandwidth	45 GHz @ −2 V bias	30 GHz @ −3 V bias

^a^ Values not available during the time of publication.

^b^ Building block not available on MPWs.

## 4 Optical Frequency Comb Generation

For the first PIC example, we present the widely tunable electro‐optic modulation‐based OFCG, reported in ref. [11], monolithically integrated on the generic InP platform. The device achieves a 20‐dB optical bandwidth of 480 GHz at 20 GHz comb‐line spacing, with optical signal‐to‐noise ratio (OSNR) exceeding 25 dB and an average −11 dBm per comb line at a 1531 nm carrier. Table 2 shows the comparison of this OFCG device with state‐of‐the‐art in InP PICs. Ref. [5] reported an OFCG based on cascaded phase modulators using an amplified recirculating loop, but the device has limited FSR tunability (0.15 GHz), owing to the constraint from the cavity resonance frequency. Ref. [6] reported gain switched laser‐based OFC, being FSR tunable (12.5 GHz) but not in wavelength. However, electro‐optic modulation based OFCGs offer tunability both in wavelength and FSR [7]. From Table 2, it can be seen that the presented OFCG chip is the most widely tunable InP OFCG PIC.

**TABLE 2:** Comparison of state‐of‐the‐art performance of integrated InP OFCG PICs.

OFCG method	20‐dB Bandwidth	FSR Tunability	Wavelength Tunability	Avg. Power per line
Amplified recirculating loop [12]	570 GHz^a^	0.15 GHz	0.24 nm	< −17 dBm^a^
Gain switched laser [13]	290 GHz	6.25 GHz	—	< −30 dBm
Cascaded EO modulators [14]	200 GHz	7 GHz	2 nm	< −20 dBm
This work	480 GHz	15 GHz	35 nm	−11 dBm

^a^ excluding the carrier.

The PIC in this work, fabricated in an MPW run using FhG‐HHI's open‐access PDK, cascades two high‐speed phase modulators (4.5 mm long PM1 and 2.25 mm long PM2) with a 600 μm on‐chip SOA in a compact 12 × 0.6 mm^2^ footprint. Facets end in deeply etched waveguides and are AR‐coated. The setup stabilizes the chip (shown in Figure 4a) at 20°C on a copper mount, uses lensed fibers for optical I/O, RF probe heads for modulator drive, and DC needles for SOA and biasing. A tunable low‐linewidth TE‐polarized external‐cavity laser provides the input carrier at 0 dBm to avoid SOA saturation. The spectra are measured with a high‐resolution optical spectrum analyzer (OSA). The modulators are biased at −9 V, with *V*_π_ of approximately 3.1 V for PM1 and 6.2 V for PM2, and are driven up to 22 dBm. Although device bandwidths exceed 30 GHz, the available RF sources limit measurements to 20 GHz. The phase offset between the RF signals is tuned to improve the comb quality and to suppress the optical carrier to the power level of the generated sidemodes.

**FIGURE 4:**
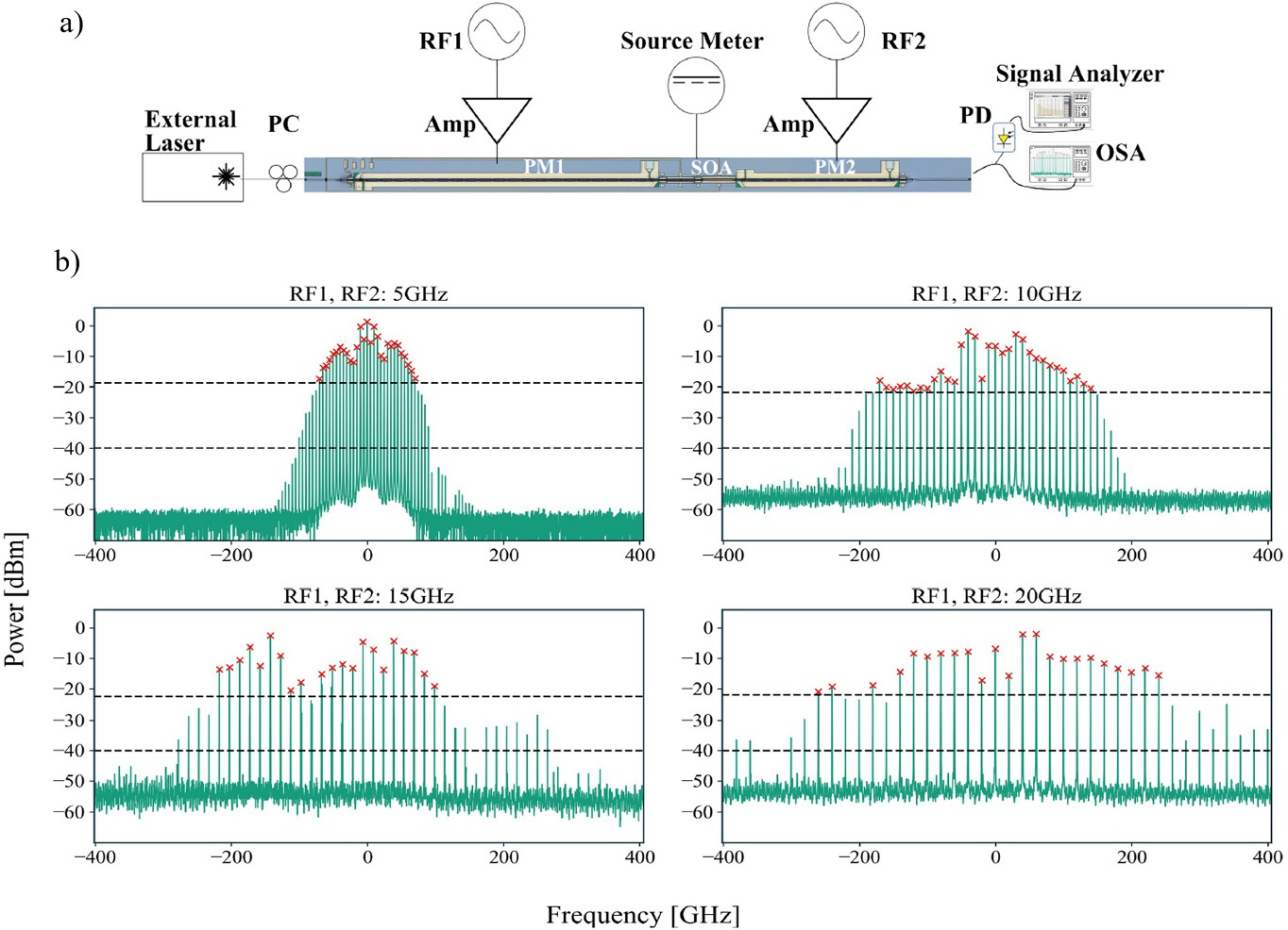
(a) Schematic of the measurement setup for OFC generation with a microscopic image of the InP PIC. (b) OFCs generated at 1531 nm carrier wavelength for different free spectral ranges (FSRs). The widest comb of 480 GHz is achieved for FSR of 20 GHz. Reproduced from ref. [11].

At 1531 nm (Figure 4b), free‐spectral‐ranges of 5, 10, 15, and 20 GHz produce OFCs with 20‐dB bandwidths of 110, 300, 315, and 480 GHz, respectively. From Figure 5, the phase noise of the fundamental frequency for each of the combs is approximately −60 dBc/Hz at minimum offset, drops below −100 dBc/Hz at 100 kHz, and falls below −120 dBc/Hz at higher offsets; a rise around 150 kHz can be attributed to the RF1 source noise.

**FIGURE 5:**
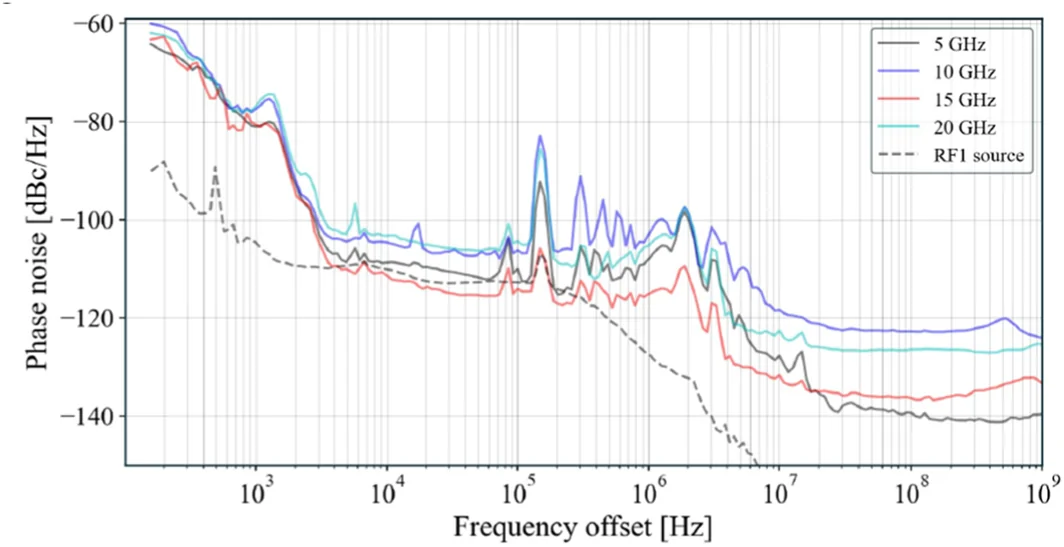
Phase noise measurement at the fundamental frequency for the combs with carrier wavelength of 1531 nm.

Table 3 lists the 20‐dB bandwidths for combs at different wavelengths, highlighting the device's wavelength tunability. This approach offers the broadest tunability in FSR and wavelength alongside high per‐line power.

**TABLE 3:** *f*_20dB_ for the generated OFCs for different carrier wavelengths and modulation frequencies.

Carrier wavelength	*f*_20dB_ (GHz)			
	5 GHz	10 GHz	15 GHz	20 GHz
1531 nm	110	300	315	480
1550 nm	80	140	165	260
1565 nm	75	120	135	140

One of the key applications for OFCG PICs is microwave photonics [7, 15], where the priority is on high optical power per line (≥ 10 dBm) and low phase noise (≤ 100 dBc/Hz at 10–100 kHz offsets). Our OFCG PIC closely approaches these stringent standards, delivering an average power per line of −11 dBm and dropping phase noise below −100 dBc/Hz at a 100 kHz offset. Drive voltage and modulator bandwidth remain the primary limitations on comb flatness and bandwidth. Future improvements will address these through device design and metallization optimizations to increase modulator bandwidth and reduce drive voltage, enabling flatter, and broader combs at lower power consumption. Integration of spot‐size converters (SSCs) will further increase the fiber‐coupled power per comb line and improve the signal‐to‐noise ratio, directly benefiting microwave photonic link budgets [16].

## 5 Monolithic Extended‐Cavity Mode‐Locked Laser

In this section, we summarize the work reported in ref. [17] of a chip‐scale, monolithic InP extended‐cavity mode‐locked laser (MLL) that achieves a record‐low repetition rate of 672 MHz under passive mode‐locking. Various on‐chip MLL structures based on active, passive, and hybrid mode locking have been demonstrated with repetition rates approaching 1 GHz, fabricated on monolithic InP [18] as well as on heterogeneous silicon/III‐V platform [19, 20, 21, 22]. Table 4 summarizes the state‐of‐the‐art results reported in the literature alongside the current work. Notably, the presented work achieves the lowest comb line spacing reported to date.

**TABLE 4:** Comparison between the presented mode‐locked laser and the state‐of‐the‐art results in the literature.

Material platform	Mode locking	FSR (MHz)	Comb bandwidth at −10 dB (nm)	Electrical linewidth (kHz)
III–V on Si [19]	Passive	1000	> 8.9	0.15
III–V on Si_3_N_4_ [20]	Passive	755.2	3.27	146
III–V on Si [21]	Passive	1009	12	0.9
III–V on Si [21]	Hybrid	1009	13	0.001
III–V on Si [22]	Active	927	0.1	—
InP [18]	Hybrid	1038.5	< 1	500
This work	Passive	672	6.7	0.5

In this work, the design leverages low‐loss passive waveguides and optimized active–passive butt joints to realize a very long on‐chip cavity without resorting to bulky fiber lasers or hybrid external cavities. The resulting RF comb is flat and densely spaced at 672 MHz, with high signal quality measured on a 40 GHz photodiode. The electrical beat tone exhibits a sub‐kHz linewidth, approximately 500 Hz at 10 dB, and a signal‐to‐noise ratio exceeding 40 dB across a 10 kHz–10 GHz span. The optical spectrum shows a 6.5 nm bandwidth at −10 dB, corresponding to approximately 1150 narrowly spaced comb lines, which enables superior resolution for dual‐comb spectroscopy and, with active mode‐locking low‐noise microwave generation.

Figures 6a,b and 7a,b provide a microscope image of the fabricated device, a wide‐span electrical spectrum of the generated pulse train, the fundamental RF tone linewidth, and the optical spectrum.

**FIGURE 6:**
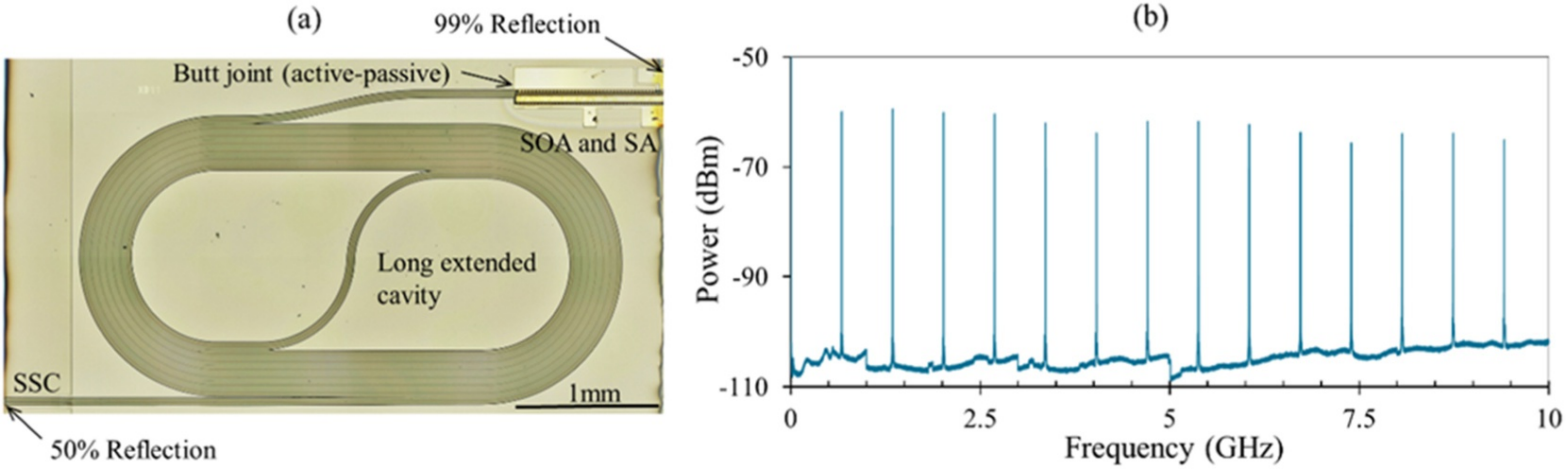
(a) Microscope image of the fabricated MLL. (b) Wide‐span electrical spectrum of the generated pulse train (100 kHz resolution bandwidth and 1 kHz video bandwidth used to obtain the RF spectra). Reproduced from ref. [17].

**FIGURE 7:**
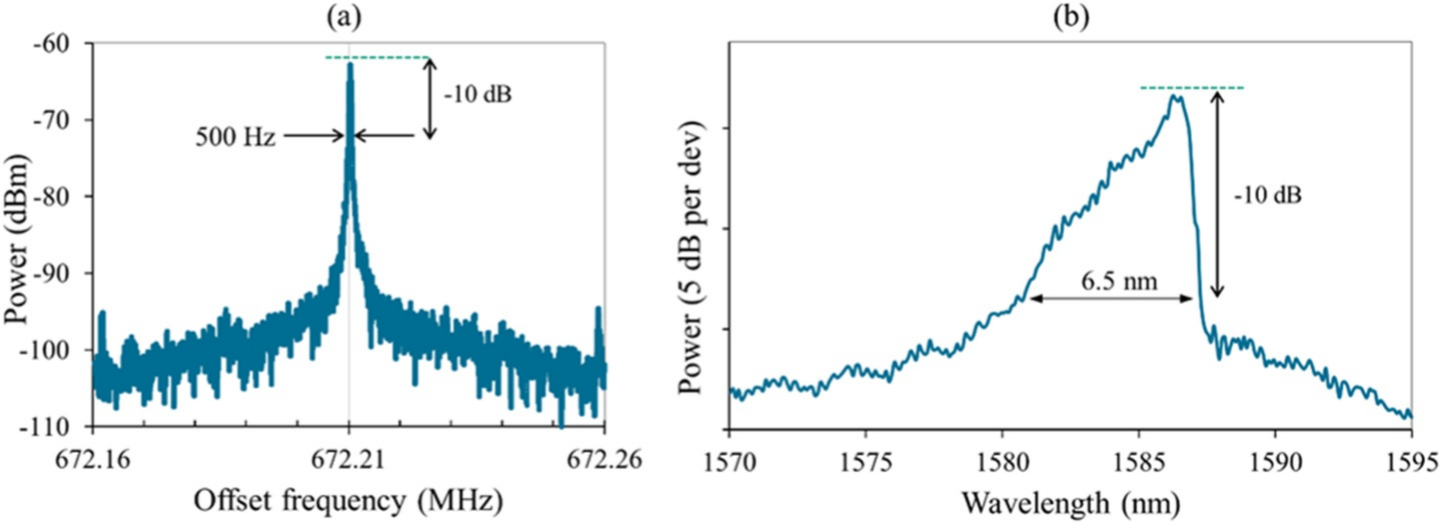
(a) Linewidth of the fundamental RF tone. (b) Optical spectrum, with a 10‐dB span of 6.5 nm, measured with a 30 p.m. resolution. Reproduced from ref. [17].

The laser integrates a 54 mm spiral InGaAsP passive waveguide and a fiber‐matched spot‐size converter with a 1000 μm SOA and a 100 μm electrically isolated saturable absorber. Forming a 64 mm cavity after facet coatings with approximately 99% reflectivity on the saturable absorber side and around 50% on the SSC side. Fabrication is performed on 3‐inch InP wafers using two‐step MOVPE with selective area regrowth, photolithography, RIE/ICP, and wet etching. The SOA and saturable absorber employ strained MQWs and low‐resistance heterostructure contacts to reduce power consumption. Characterization at 15°C shows a threshold current of approximately 75 mA with the saturable absorber at zero bias, and more than 0.4 mW fiber‐coupled power at 180 mA SOA current. The operating temperature is set to 15°C instead of the usual 20°C in order to optimize the device performance. Passive mode‐locking is achieved with 1 V reverse bias on the saturable absorber and 180 mA SOA current, yielding the flat RF comb and narrow fundamental tone described above; the optical comb was measured with a 30 pm‐resolution OSA.

For dual‐comb spectroscopy [23], system requirements dictate a broad optical bandwidth (> 5 nm at −10 dB) alongside an ultra‐stable comb spacing characterized by a sub‐kHz RF linewidth to prevent overlapping spectral aliases and preserve long‐term signal coherence. The demonstrated MLL PIC successfully achieves a 6.5 nm optical bandwidth at −10 dB, a 500 Hz RF linewidth, and an RF SNR exceeding 40 dB, placing it competitively alongside modern on‐chip frequency tools for high‐resolution spectroscopic applications. The primary architectural limitations of the current design are its modest fiber‐coupled output power (> 0.4 mW at 180 mA) and its fixed passive mode‐locking regime. Future developments will explicitly target higher output power by integrating a booster SOA and shortening the saturable absorber length, further enhancing the device's utility in spectroscopic systems and low‐noise microwave photonics.

## 6 Monolithic Tunable Narrow‐Linewidth Ring Laser

The last PIC example is a monolithic InP ring‐resonator tunable laser (reported in refs. [24, 25]) designed as a local oscillator for coherent optical receivers, where tuning range, side‐mode suppression ratio, linewidth, and output power are critical contributors to system performance and bit‐error rate. Unlike heterogeneously or hybrid‐integrated lasers that can achieve sub‐kHz linewidths but require complicated co‐integration, monolithic InP enables seamless integration with high‐speed coherent receiver PICs [26]. Prior monolithic approaches include distributed Bragg reflector (DBR) lasers [27] with approximately 10 kHz linewidth but very limited tuning, sampled grating DBR [28] with around 70 kHz linewidth and complex thermal isolation, and actively tuned ring lasers [29] with roughly 110 kHz linewidth but low power due to doped‐waveguide loss.

Similar to distributed Bragg reflector (DBR) lasers and sampled grating DBR (SGDBR) lasers, the presented tunable ring laser utilizes the Vernier effect, with cascaded ring resonators to achieve wide wavelength coverage. Alternatively, intracavity multichannel interferometer (MCI) lasers achieve wavelength selection through cascaded asymmetric Mach–Zehnder interferometers for tuning with different FSRs. Recent demonstrations [30, 31] have shown tuning ranges exceeding 50 nm with SMSRs above 40 dB, positioning MCI lasers as viable candidates for widely tunable InP laser architectures.

Our approach minimizes cavity loss by using low‐loss passive waveguides for two thermally tuned ring resonators of approximately 400 μm radius each, together with a Sagnac‐loop total reflector, all coupled via 50:50 multimode interference couplers (MMIs). A 400 μm SOA provides gain, and a 200 μm phase section allows fine tuning by current injection or electro‐optic control. The Vernier FSR is approximately 90 nm. Light is extracted through an MMI to a spot‐size converter for fiber coupling. The device is fully compatible with HHI MPW foundry runs, fabricated on 3‐inch InP with contact lithography, lift‐off, etching, and MOVPE regrowth to form butt‐joints between active and passive regions; the SOA utilizes MQWs with a photoluminescence peak around 1568 nm. A photograph of the fabricated tunable laser PIC is shown in Figure 8.

**FIGURE 8:**
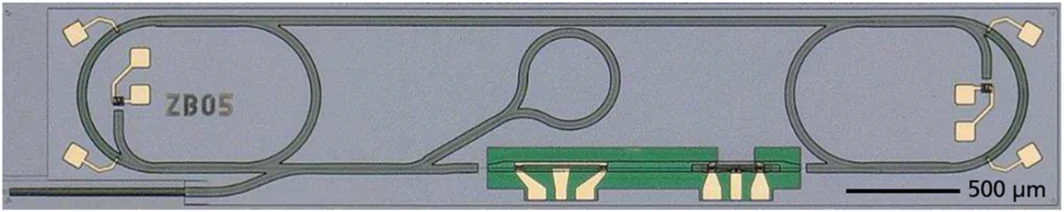
Photograph of the fabricated tunable laser PIC. Reproduced from ref. [24].

From Figure 9, it can be seen that the fiber‐coupled output power reaches 4.9 mW at 150 mA SOA current and up to approximately 6 mW at 220 mA, with visible kinks from mode hopping. Single‐mode operation spans 1559–1602 nm, giving more than 40 nm of tuning, narrower than the Vernier FSR due to the SOA's gain profile favoring modes near its peak and inducing mode competition. Heater power is around 50 mW per ring, and the phase section uses roughly 5 mW in reverse bias. The side‐mode suppression ratio remains above 36 dB across the tuning range. Linewidth is assessed via delayed self‐heterodyne using an 11 km fiber and an 80 MHz acousto‐optic modulator. Measured 3‐dB beat widths of approximately 420 kHz at 50 mA and 520 kHz at 100 mA reflect total noise. Frequency‐noise spectral density measured with an optical frequency noise analyzer shows a frequency noise floor of 2616 Hz^2^/Hz, yielding an intrinsic linewidth of 8.2 kHz.

**FIGURE 9:**
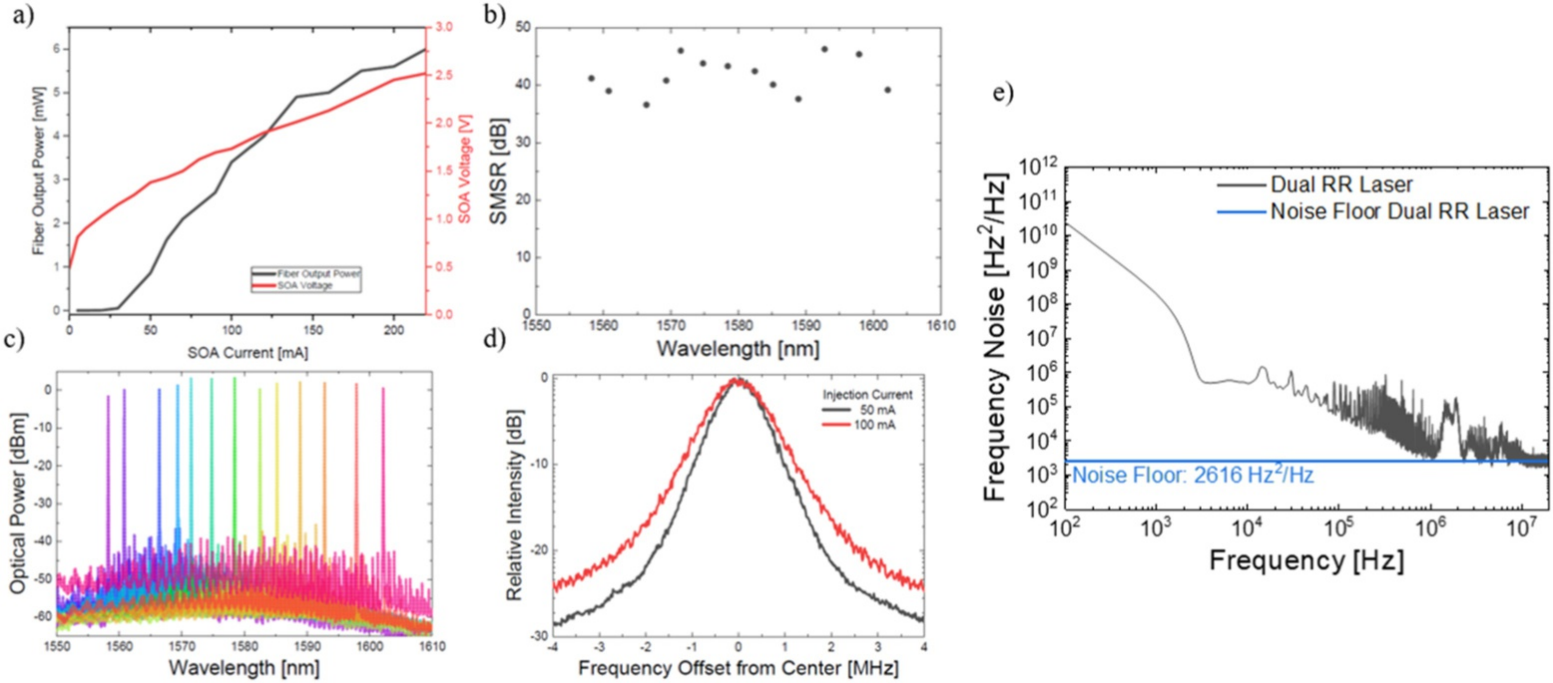
(a) Voltage and optical power coupled into the fiber in dependence of injection current. (b) Side mode suppression ratio in dependence of wavelength. (c) Spectrum of the laser for a SOA forward current of 150 mA, the resolution bandwidth was set to 0.03 nm. (d) Generated RF signals of the delayed self‐heterodyne measurement setup. (e) Measured optical frequency noise. Reproduced from ref. [24].

Table 5 summarizes the state‐of‐the‐art across available architectures. For completeness, the monolithic optical injection locking (MOIL) laser [32] which has the narrowest linewidth reported on InP has also been included despite the corresponding laser not being wavelength‐tunable.

**TABLE 5:** State‐of‐the‐art comparison of low linewidth InP tunable lasers.

	Output power	Tuning range	SMSR	Linewidth
DBR [27]	18 mW	—	54 dB	10 kHz
SGDBR [28]	50 mW	41 nm	50 dB	70 kHz
Active ring [29]	1 mW	34 nm	50 dB	110 kHz
MCI filter [30]	64 mW	56 nm	49 dB	90 kHz
MCI filter [31]	6.6 mW	51 nm	40 dB	16 kHz
MOIL [32]	11 mW	—	50 dB	0.98 kHz
Passive ring (This work)	6 mW	41 nm	36 dB	8.2 kHz

For high‐speed coherent communications, the local oscillator demands are defined by broad single‐mode tuning ranges (> 35 nm to completely span the telecommunication C‐band), strong SMSRs (> 40 dB), and narrow intrinsic linewidths (< 100 kHz) for advanced PAM/QAM modulation formats [33]. The demonstrated tunable ring laser bridges these application gaps by offering a 40 nm single‐mode tuning range and an 8.2 kHz intrinsic linewidth, while achieving a competitive SMSR above 36 dB across the full span with up to 6 mW of fiber‐coupled output power. The SMSR and output power can be further optimized through refined ring path lengths and coupling ratios and through the integration of booster SOAs.

## 7 Packaging and Evaluation

Photonic integration for manufacturable systems extends beyond the initial chip fabrication. Customers new to PICs often underestimate the complexity and cost of testing and packaging, which can account for the majority of total manufacturing cost and are typically not covered by standard foundry services. Incorporating additional suppliers introduces logistical challenges and slow feedback loops, delaying time‐to‐market. To address this gap, FhG‐HHI complements its foundry service [34] with standardized packaging options that offer short cycle times and consistent measurement workflows. This vertically integrated approach lowers the entry barrier for customers and enables prototypes at high TRLs.

Based on high‐level standards for PIC layouts, HHI provides design templates compatible with the thermally controllable RFconnect Module [35] as shown in Figure 10a,b,d–h. RFconnect supports multiple chip sizes and offers up to 42 DC channels, eight optical I/O, and eight RF I/O channels for applications up to 40 GHz. A corresponding RF evaluation board with a clamping mechanism facilitates assembly handling and enables module substitution without rework. A compatible source meter, the PIConnect Mainboard [36] shown in Figure 10c, is specifically designed to drive active building blocks in the PIC, enabling instantaneous measurements and fast prototyping. Together, these offerings implement open testing standards [37] to streamline cross‐vendor workflows and improve repeatability.

**FIGURE 10:**
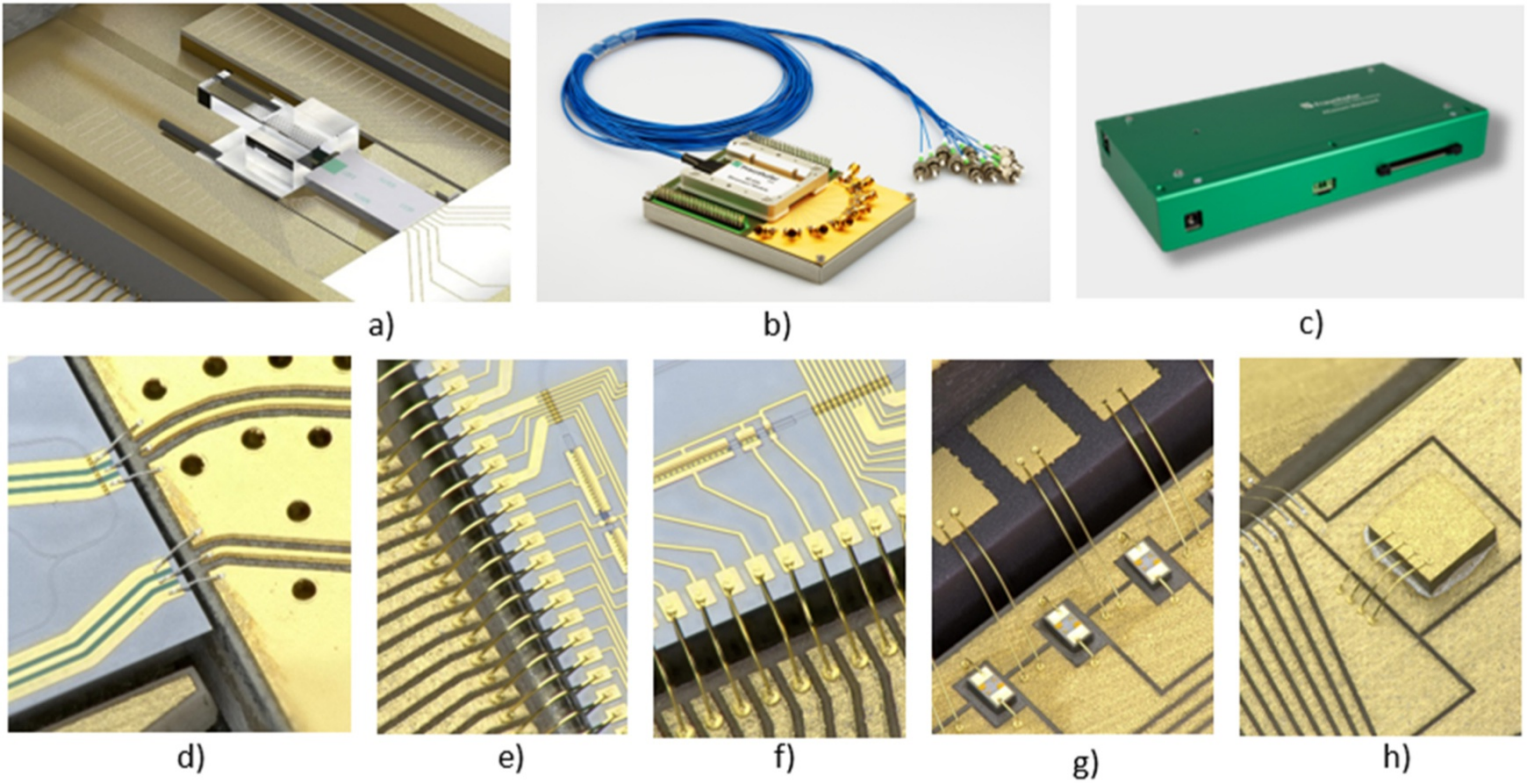
(a) Rendered interior of RFconnect module; (b) RFconnect module clamped on RF evaluation board; (c) PIConnect mainboard; and (d)–(h) close‐ups of assembly process showing RF bonds, DC bonds, electrostatic discharge (ESD) protection, and the thermistor.

Although scaling the emitter PICs, high integration complexity and channel density introduce thermal and fabrication challenges, which can be resolved through targeted engineering strategies. Increased thermal loads can be managed through optimizing material compositions, integrating dedicated heat sinks, and substrate thinning. Additionally, precise fabrication control is needed to ensure uniform performance across large dies. Although the presented emitter PICs rely on bare‐die cooling, transitioning to package‐level thermal management with the presented packaging solution is a viable path forward for next‐generation devices.

## 8 Conclusion and Outlook

We have presented an update on an open‐access, PDK‐enabled FhG‐HHI's InP foundry platform that integrates high‐performance active and passive functions and supports scalable pathways from MPW prototypes to customized modules and volume production. The platform demonstrates high‐speed Mach–Zehnder modulators operating at 80 GBaud with low drive voltage, a monolithic tunable ring laser with more than 40 nm tuning range and an intrinsic linewidth of 8.2 kHz, a monolithic extended‐cavity mode‐locked laser achieving a 672 MHz repetition rate and more than one thousand lines over approximately 6.5 nm, and a widely tunable electro‐optic comb generator with flexible spacing between 5 and 20 GHz and broad wavelength agility. The demonstrated all‐electrical testing provides a pathway to wafer‐scale predicing testing, enabling yield control and reducing cost.

Future platform developments target several performance dimensions simultaneously. Modulator bandwidth will be increased and drive voltage further reduced through device design and metallization optimizations, enabling higher data rates at lower power consumption. Integrating SSCs to the comb chip will increase output power per comb line and improves the SNR, whereas the modulator performance improvements increase the bandwidth of the comb. Future improvements for the MLL target higher output power by integrating a booster SOA and shortening the saturable absorber, further enhancing utility in spectroscopic systems and low‐noise microwave photonics. The narrow‐linewidth tunable laser performance will be enhanced through refined ring path lengths and coupling ratios, integration of booster SOAs, and yielding higher side‐mode suppression ratios and greater output power.

## Author Contributions

**Durvasa Y. Gupta:** conceptualization, methodology, data curation, investigation, validation, formal analysis, visualization, project administration, writing – original draft, writing – review and editing. **Axel Schoenau:** conceptualization, methodology, investigation, writing – review and editing, data curation, validation, visualization. **Hendrik Boerma:** investigation, methodology, data curation, writing – review and editing, validation, visualization. **Shahram Keyvaninia:** writing – review and editing, methodology, investigation, data curation, validation, visualization. **Patrick Runge:** conceptualization, supervision, project administration, funding acquisition, writing – review and editing.

## Data Availability

The data that support the findings of this study are available from the corresponding author upon reasonable request.
